# Ioncopy: a novel method for calling copy number alterations in amplicon sequencing data including significance assessment

**DOI:** 10.18632/oncotarget.7451

**Published:** 2016-02-17

**Authors:** Jan Budczies, Nicole Pfarr, Albrecht Stenzinger, Denise Treue, Volker Endris, Fakher Ismaeel, Nikola Bangemann, Jens-Uwe Blohmer, Manfred Dietel, Sibylle Loibl, Frederick Klauschen, Wilko Weichert, Carsten Denkert

**Affiliations:** ^1^ Institute of Pathology, Charité University Hospital, Berlin, Germany; ^2^ German Cancer Consortium (DKTK), partner sites Berlin, Heidelberg and Munich, Germany; ^3^ Institute of Pathology, University Hospital, Heidelberg, Germany; ^4^ Institute of Pathology, Technical University Munich (TUM), Munich, Germany; ^5^ National Center for Tumor Diseases (NCT), Heidelberg, Germany; ^6^ Department of Pathology, Center for Integrated Diagnostics (CID), Massachusetts General Hospital/Harvard Medical School, Boston, USA; ^7^ Department of Gynecology, Charité University Hospital, Berlin, Germany; ^8^ Interdisciplinary Breast Center, Charité University Hospital, Berlin, Germany; ^9^ German Breast Group (GBG), Neu-Isenburg, Germany

**Keywords:** targeted sequencing, amplicon sequencing, semiconductor sequencing, copy number alterations, breast cancer

## Abstract

Recently, it has been demonstrated that calling of copy number alterations (CNAs) from amplicon sequencing (AS) data is feasible. Most approaches, however, require non-tumor (germline) DNA for data normalization. Here, we present the method Ioncopy for CNA detection which requires no normal controls and includes a significance assessment for each detected alteration.

Ioncopy was evaluated in a cohort of 184 clinically annotated breast carcinomas. A total number of 252 amplifications were detected, of which 183 (72.6%) could be validated by a call of an additional amplicon interrogating the same gene. Moreover, a total number of 33 deletions were found, whereof 27 (81.8%) could be validated. Analyzing the 16 most frequently amplified genes, validation rates of over 89% could be achieved for 11 of these genes. 11 of the top 16 genes showed significant overexpression in the amplified tumors. 89.5% of the *HER2*-amplified tumors were *GRB*7 and *STARD3* co-amplified, whereas 68.4% of the *HER2*-amplified tumors had additional *MED1* amplifications. Correlations between CNAs measured by amplicons in *HER2* exons 19, 20 and 21 were strong (all *R* > 0.93). AS based detection of *HER2* amplifications had a sensitivity of 90.0% and a specificity of 98.8% compared to the gold standard of *HER2* immunohistochemistry combined with *in situ* hybridization.

In summary, we developed and validated a novel method for detection and significance assessment of CNAs in amplicon sequencing data. Using Ioncopy, AS offers a straightforward and efficient approach to simultaneously analyze gene amplifications and gene deletions together with simple somatic mutations in a single assay.

## INTRODUCTION

Inherited genetic variation and acquired genomic aberrations are constitutive for cancer initiation and cancer progression. In the era of cancer precision medicine, monitoring of clinically relevant genetic alterations is important to stratify patients for targeted therapies. In addition to ”conventional” mutations such as point mutations, small insertions and deletions, clinically relevant genetic alterations include macro-aberrations such as amplifications, deletions or translocations. In particular, *HER2* amplifications in breast cancer exemplify the important biological role and clinical utility of copy number variations/alterations (CNVs/CNAs) in oncological therapy. *HER2* testing and anti-*HER2* treatment revolutionized breast cancer care in 1998, when the FDA approved Herceptin for the treatment of metastatic breast cancer following the success of the first phase III clinical trial [1]. Subsequently, Herceptin was also approved for early breast cancer in the adjuvant setting after completion of the NSABP/NCCTG and HERA trials [2, 3]. Furthermore, a recent pan-cancer analysis of more than 3,000 TCGA tumors resulted in two top classes either dominated by somatic mutations (M class) or dominated by somatic copy number alterations (C class) [4]. The C class included almost all breast cancers and almost all high-grade serous ovarian cancers. For breast cancer, this assignment is supported by the fact that the number of recurrently mutated genes is low [5], while copy number alterations (together with gene expression data) built the basis of the recent METABRIC breast cancer classification in ten internal clusters [6]. Complementing *HER2*, the landscape of all gene amplifications and deletions may represent a collection of promising candidates for future biomarkers and therapeutic targets in breast cancer.

There is a multitude of methods to detect germline CNVs or somatic CNAs in cancer cells [7]. These methods can be classified in different ways: Firstly, there are basically two types of technologies, hybridization based and next-generation sequencing (NGS) based approaches. Secondly, there are technologies interrogating the whole genome such as comparative genomic hybridization (CGH), SNP arrays or whole genome sequencing as opposed to technologies that analyze selected regions of the genome. These targeted approaches range from whole exome sequencing via sequencing of a smaller number of selected gene regions to the traditional locus-specific methods such as fluorescence or silver- enhanced *in situ* hybridization (FISH or SISH). Thirdly, it is important to distinguish between the ISH based methods that allow cell-specific assessment of CNAs under the microscope and all other methods that are based on many-cell-averages and possibly include genetically different cells, such as cancer cells, normal cells and differing subclones of cancer cells.

In recent years, targeted NGS has been established for routine molecular diagnostics of cancers to interrogate clinically actionable genetic aberrations. This implementation was driven by the advances in personalized oncology with both a growing number of actionable genetic targets in a single tumor and a growing number of patients being investigated for these targets. In addition to the detection of somatic mutations, it was shown that the detection of CNAs from targeted sequencing data is generally feasible [8, 9]. Methodically, CNA calling in amplicon sequencing (AS) data relies on calculation of the amplicon coverages and the detection of coverage outliers after a suitable normalization. To this end, most of the current algorithm require sequencing of paired tumor and normal DNA samples [10] or utilize a normal DNA reference sample for normalization [8, 11]. However, in routine diagnostics normal control tissue is not always available, particularly when it comes to genotyping of small biopsies. In addition, sequencing normal tissue in parallel would double the costs for diagnostic AS applications per case. Moreover, most of the current algorithms include neither significance assessment nor correction for multiple hypotheses testing.

To overcome these limitations, we developed and evaluated Ioncopy, a new algorithm to detect CNAs from AS data that is freely available as R package from the CRAN repository. As input, the algorithm uses sequencing data of cohort of tumors and does not require normal DNA controls. The guiding idea is to estimate a null distribution of copy numbers using outlier-robust statistics and assess the significance of CNAs by comparison with this null distribution. In this way, p-values are obtained for each amplicon in each tumor that are subsequently corrected for multiple hypothesis testing.

We tested Ioncopy in AS data obtained with a 154-amplicon-panel that was designed to include the most important simple somatic mutations and gene amplifications in breast cancer. Using this panel, a clinical cohort of 184 breast carcinomas was sequenced and data were analyzed for the detection of CNAs using the new algorithm. The performance of Ioncopy was evaluated by (i) comparing the detected *HER2* CNAs with the *HER2* status determined by the gold standard of immunohistochemistry (IHC) and ISH [12], (ii) comparing the CNs detected by different amplicons interrogating the same gene, (iii) analyzing the overall CNA landscape of all 48 genes covered by the panel and (iv) correlating the Ioncopy CNA calls with the tumor RNA expression of the corresponding gene.

## RESULTS

### Detection of copy number alterations

**Figure 1:**
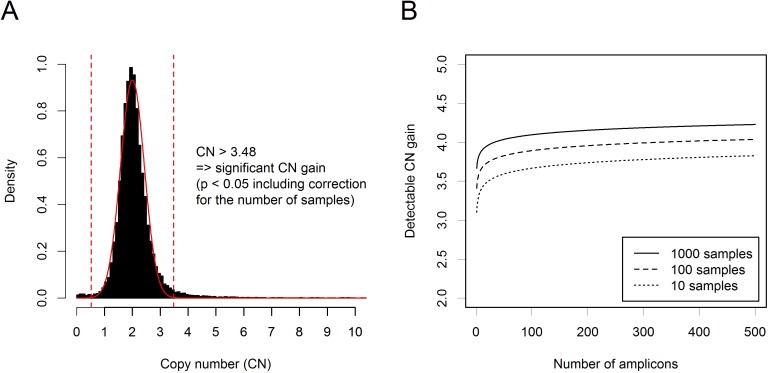
Ioncopy algorithm for detection and significance assessment of CNAs in amplicon sequencing data **A.** Distribution of CNs (184 tumors, 152 amplicons) with fitted curve of a normal distribution centered at CN = 2. A threshold of CN = 3.48 corresponds to significant copy number gains after multiple testing correction for the tumors. A threshold of CN = 3.99 (not shown) corresponds to highly significant copy number gains after multiple testing correction for tumors and amplicons. **B.** Effect of correction for multiple testing on the detection limits for CN gain. Simulation analysis varying the number of samples between 10 and 1000 and the number of amplicons between 1 and 500. For all simulated situations, CN gains of 5 and more can be detected with high sensitivity and specificity. Detection of CN gains of 4 is feasible in some situations, for example when the number of genes under investigation is low.

Targeted DNA-sequencing of of 184 fresh-frozen breast cancer tissues was executed using a custom-designed panel including 154 amplicons. 152 amplicons had a sufficient sequencing depth (mean coverage ≥ 100) and were included in the analysis. Copy numbers (CNs) were estimated after sample-normalization and subsequent amplicon-normalization as described in the Methods section. The resulting distribution of CNs is shown in Figure 1A. The core of the distribution, excluding outliers that are expected to correspond to CNAs, could be well approximated by a normal distribution centered at CN = 2. Thus, the significance of CNAs could be assessed using the estimated normal distribution as null distribution.

The resulting p-values required correction for multiple hypotheses testing, as each sample and each amplicon are tested for CNAs. When analyzing a single amplicon (multiple testing correction for samples), significant amplifications corresponded to CN > 3.48, while significant losses corresponded to CN < 0.52. When analyzing the whole cohort (multiple testing correction for samples and amplicons), significant amplifications corresponded to CN > 3.99, while significant losses corresponded to CN < 0.01. A simulation analysis was carried out to investigate the effect of multiple testing corrections on the detection limit for CNAs. To this end, we varied the number of samples as well as the number of amplicons and estimated the corresponding detection thresholds for CN gains (Figure 1B). The results indicate that detection of CN gains of 5 and more is feasible at high sensitivity and specificity over a wide range of numbers of samples and numbers of genes. Additionally, sensitive and specific detection of CN gains of 4 may also be possible, provided that the number of genes under consideration is low.

### Calling gene amplifications

**Figure 2:**
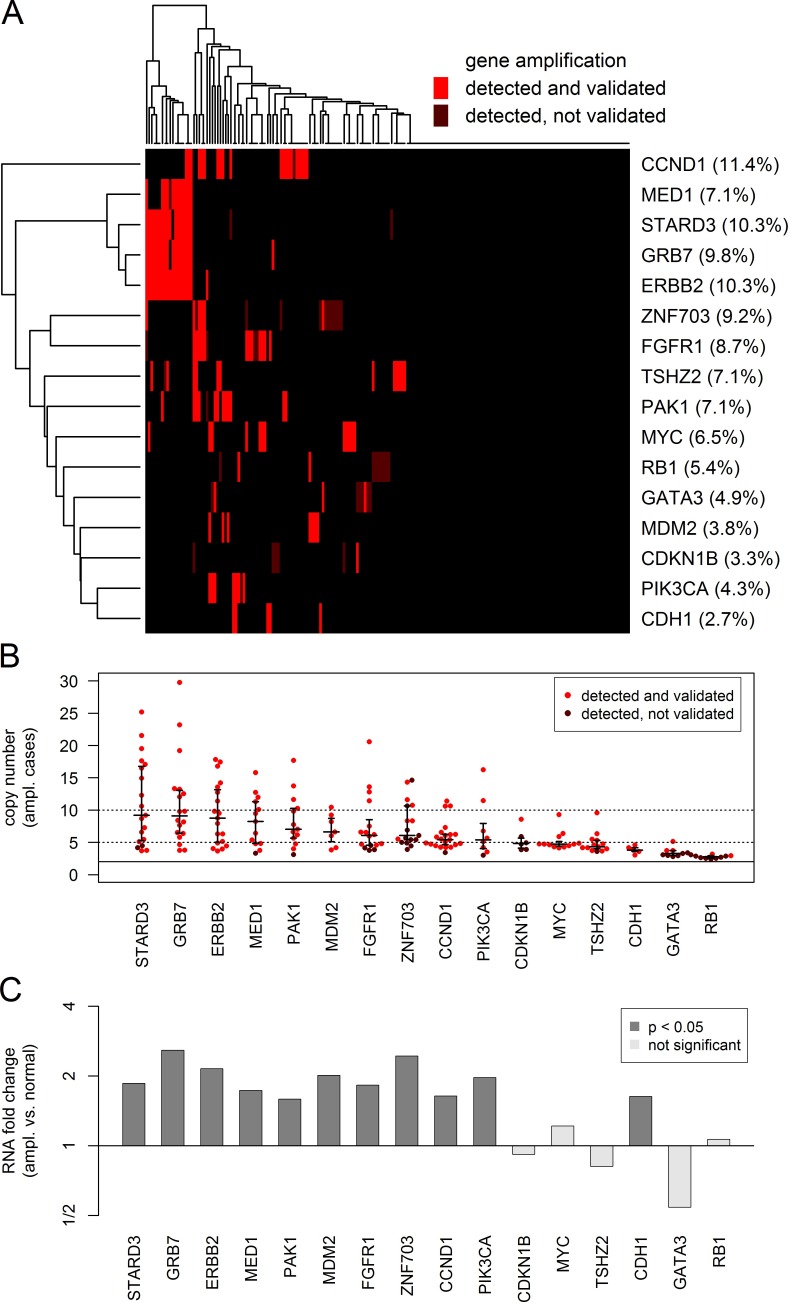
Analysis of the CN gains in 16 genes that were amplified in at least 5 tumors CN gains were considered as detected if highly significant (multiple testing correction for tumors and amplicons) for at least one interrogating amplicon and as validated if significant (multiple testing correction for tumors) for a second additional amplicon. **A.** Heatmap showing the global pattern of gene amplifications and the percentage of amplified tumors for each of the genes. **B.** Beeswarm plot showing the CN gains in the amplified tumors (red dots) and the 25%, 50% and 75% quantiles of the corresponding distribution (black lines). C Barplot showing the RNA expression changes between amplified and unamplified tumors. Significant RNA overexpression 11 of the 16 genes in the amplified tumors.

Overall, a total of 252 (2.9% of all genes and tumors) gene amplifications affecting 39 genes were detected at high significance level (Bonferroni correction for samples and amplicons). Out of these 183 (72.6%) could be validated by a call of a second amplicon. We executed a more detailed analysis of the top 16 genes that were amplified in at least 5 samples (Figure 2). For 11 genes out the top genes, 89% or more of the detection calls could be validated by a call of a second amplicon. Lower validation rates were obtained only for *ZNF703* (35%), *GATA3* (33%), RB1 (20%) and *CDKN1B* (17%). For *GATA3*, all 9 amplifications were detected by a single amplicon in exon 5. For *RB1*, all amplifications were detected by a single amplicon located in exon 3. However, although *GATA3* was covered by 3 and *RB1* by 11 amplicons, only a few of the detected amplifications could be validated. Particularly, the amplicons in exon 5 of *GATA3* and in exon 3 of *RB1* appeared to be prone to false positive detection of gene amplifications and should be excluded. The situation was different for *CDKN1B* and *ZNF703* that are located in GC-rich regions of the genome. These might be true positive detections that are difficult to validate, as for each of these genes only two amplicons were available and one of the available amplicons had a low coverage (mean coverage 619 and 1343) compared to the average coverage of 4330 of the whole cohort.

The landscape of the detected gene amplifications is shown in Figure 2A. The heatmap shows the most frequently amplified genes and tumors in the left top corner. *HER2* clustered tightly together with *GRB7* and *STARD3*, all three genes being located in a core *HER2* amplification region of about 100,000 bp. Additionally, two more genes located in the region 17q12-21 were interrogated by the panel, *MED1* located about 300,000 bp upsteam from *HER2* towards the centromere and *TOP2A* located about 700,000 bp downsteam from *HER2* towards the telomere. Overall, two amplifications of *TOP2A* were detected, one of which could be validated (data not shown). Out of the 19 tumors with detected and validated *HER2* amplifications, 17 (89.5%) harbored amplified *GRB7*, 17 (89.5%) amplified *STARD3*, 13 (68.4%) amplified *MED1* and 2 (10.5%) amplified *TOP2A*.

The detected CNs of the gene amplifications are shown in Figure 2B. Some of the gene amplifications (*n* = 47, 22.8%) resulted in high CNs ≥ 10, whereas most of the detected gene amplification had intermediate dose (*n* = 70, 34.0%) or resulted in moderate CNs < 5 (*n* = 89, 43.2%). Differential RNA expression between amplified and unamplified tumors was found for 11 of the 16 top genes (Figure 2C).

### Analysis of the detected HER2 amplifications

**Figure 3:**
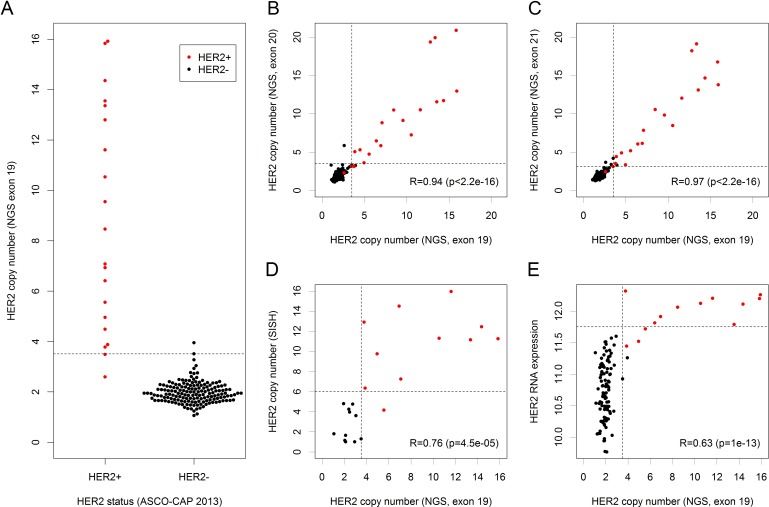
Detection and analysis of *HER2* amplifications *HER2*+ status of tumors was determined according to the 2013 ASCO-CAP recommendations (red dots = *HER2*+ tumors, black dots = *HER2*- tumors). We analyzed the amplifications detected by the amplicon in exon 19 of *HER2* and considered a CN gain as detected if significant after correction for the number of tumors. **A.** *HER2* CNs detected by Ioncopy (amplicon in exon 19) including thresholds for the detection of gains (CN = 3.51, dashed line). Compared to ASCO-CAP as gold standard, Ioncopy had a sensitivity of 90.0% and a specificity of 98.8%. **B.** Correlation analysis of CNs detected by the amplicon in exon 19 and by the amplicon in exon 20 (R = Pearson correlation coefficient). **C.** Correlation analysis of CNs detected by the amplicon in exon 19 and by the amplicon in exon 21. **D.** Correlation analysis of *HER2* CNs detected by Ioncopy (amplicon in exon 19) with *HER2* CNs detected by SISH. **E**. Correlation analysis of *HER2* CNs detected by Ioncopy (amplicon in exon 19) and *HER2* RNA expression. All ten tumors with high *HER2* RNA expression (≥ 11.75) were both *HER2*+ according to ASCO-CAP and *HER2*-amplified according to Ioncopy.

Our sequencing panel comprised three amplicons interrogating *HER2* located in exons 19, 20 and 21. CNs detected by the amplicon in exon 19 were compared with *HER2* status determined according to the 2013 ASCO-CAP guidelines (Figure 3A). A *HER2* gene amplification was called in 20 tumors with 16 out of these (90.0%) being *HER2*+ according to ASCO-CAP. Compared to the gold standard of ASCO-CAP, Ioncopy had a sensitivity of 90.0%, a specificity of 98.8% and an overall agreement of 97.8% for determination of *HER2* status. CNs detected by the amplicons in exon 20 and exon 21 correlated strongly with the CNs detected by the amplicon in exon 19 (*R* = 0.94 and *R* = 0.97, Figure 3B and 3C). The strong correlations of CNs resulted in almost identical calls for *HER2* amplifications: 17 amplifications were called by all three amplicons, four amplifications were called by two amplicons and a single amplification was called by only one amplicon. Furthermore, we evaluated the degree of *HER2* amplification in a subcohort of 11 *HER2*+ and 10 *HER2*- tumors using SISH and found a strong correlation of *R* = 0.76 between *HER2* CNs detected by Ioncopy and CNs detected by SISH (Figure 3D). Finally, for the tumors for which genome-wide expression data were available, a correlation analysis of *HER2* CNs and *HER2* RNA expression was carried out (Figure 3E). Interestingly, all ten tumors with high *HER2* RNA expression (≥ 11.75) were both *HER2*+ according to ASCO-CAP and *HER2*-amplified according to Ioncopy.

### Calling gene deletions

**Figure 4:**
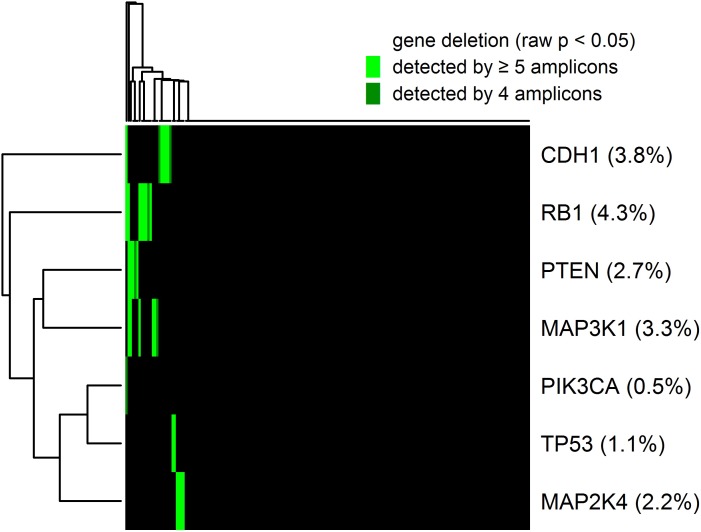
Heatmap showing the global pattern of gene deletions and the percentage of deleted tumors for each of the genes CN losses were considered as detected if significant (raw *p* < 0.05) for at least four interrogating amplicons and as validated if significant (raw *p* < 0.05) for a fifth additional amplicon. Deletions were detected in in *RB1* (8 tumors), *CDH1* (7 tumors), *MAP3K1* (6 tumors), *PTEN* (5 tumors), *MAP2K4* (4 tumors), *TP53* (2 tumors) and *PIK3CA* (1 tumors).

For the detection of gene deletion, application of the same method that was used before for the detection of gene amplifications was not feasible because the thresholds would be CN < 0.52 for significant (multiple testing correction for tumors) gene losses and CN < 0.014 for highly significant (multiple testing correction only tumors and amplicons) gene losses. Thus, the method used before lacked sensitivity for the detection of gene loss, as all heterozygous deletions and - taking into account contamination by normal tissue - many homozygous deletions would be below the detection threshold. Therefore, we used a different algorithm for the detection of gene by demanding *p* < 0.05 for the raw p-values without multiple testing correction (corresponding to CN < 1.30) for at least four amplicons. Overall, we detected a total of 33 (0.4%) gene deletions affecting 7 genes that were called by at least four amplicons. Out of these 27 (81.8%) could be validated by the significant (raw *p* < 0.05) call of a fifth amplicon. The landscape of the detected gene deletions is shown in Figure 4. Deletions were detected in in *RB1* (8 tumors), *CDH1* (7 tumors), *MAP3K1* (6 tumors), *PTEN* (5 tumors), *MAP2K4* (4 tumors), TP53 (2 tumors) and *PIK3CA* (1 tumors).

Gene expression changes between tumor with and without gene deletions were negative for *MAP2K4* (fold change = −2.37, *p* = 0.0099), *PTEN* (fold change = −1.71, *p* = 0.10) and *CDH1* (fold change = −1.59, *p* = 0.22), but close to one for *RB1* (fold change = 1.08, *p*=0.62). For *TP53* and *PIKCA* statistical analysis was impossible, as in both cases only one deleted tumor was investigated by whole-genome expression analysis, whereas *MAP3K1* was not represented by the microarray. We are aware that the p-values for *CDH1* and *PTEN* were only borderline significant. However, the numbers of deleted tumors were low and correlations between CNs estimated by Ioncopy and the gene expression levels were significant for *MAP2K4* (*R* = 0.45, *p* = 6.3e-07), *CDH1* (*R* = 0.23, *p* = 0.015) and *PTEN* (*R* = 0.19, *p* = 0.042).

### Analysis of normal tissues

Finally, we analyzed a cohort of 16 normal breast tissues using the same threshold for CNA calling as in the tumor cohort. Overall, we detected five gene amplifications, whereof one could be validated by the call of a second amplicon. No gene deletions were detected in the normal tissue cohort. Therefore, based on the hypothesis that no (or very few) CNAs occur in normal tissues, the following lower bounds for the specificity of Ioncopy can be obtained: at least 99.3% for the analysis mode without validation and at least 99.9% for the analysis mode with validation by an additional amplicon.

Interestingly, all detected CNAs occurred in a single sample (6% of the samples) while no CNAs were detected in the remaining samples (94% of the samples). Amplifications were called in the genes *RB1*, *EGFR*, *TLR4*, *MDM2* and *CDKN1B* (interrogated by 10, 4, 3, 3 and 2 amplicons). Only one of these findings (*MDM2*, CN = 4.6, *p* = 7.8e-10) could be validated by the call of an additional amplicon. In accord with the negative validation results, we expect at least some of these calls to be false positives possibly caused by a technical problem with the sample. However, except a sole exception, all amplification calls were filtered out by running Ioncopy in the validation mode. In summary, the results from the analysis of normal tissues support the assertion that Ioncopy is a highly specific method for calling copy number alterations in AS data.

## DISCUSSION

Ioncopy is a novel fast and easy-to-use algorithm to detect CNAs from AS data without normal controls. An implementation is freely available as R package ioncopy from the CRAN repository. While the general feasibility to detect CNAs using this kind of data has been demonstrated before [8, 9], to our knowledge this algorithm is the first that includes a significance assessment for each of the detected changes. Unlike the majority of other algorithms, Ioncopy does not depend on normal DNA controls, but estimates a null distribution from CNs in a tumor cohort. In routine diagnostics, there are several difficulties connected with the use of normal DNA controls: Often, normal control tissue is not available, particulary when a single small tumor biopsy of a patient has to be genotyped. In addition, sequencing of paired normal tissues would double the already considerable costs for targeted deep sequencing. Finally, in some countries there are legal issues connected with sequencing of normal DNA and putative inadvertent detection of clinically relevant germline aberrations.

A total number of 252 gene amplifications affecting 39 genes were found, whereof 183 (72.6%) could be validated by a call of a second amplicon interrogating the same gene. Analyzing the 16 top amplified genes, validation rates were higher than 89% for 11 of these genes. Furthermore, 11 of the top 16 genes showed significant overexpression in the amplified tumors compared to the unamplified tumors. A detailed analysis of the 17q12 region showed that 89.5% of the *HER2*-amplified tumors were also *GRB7* and *STARD3* amplified, whereas 68.4% of the *HER2*-amplified tumors were *MED1* amplified. The rates of co-amplifications are in good agreement to those reported in the literature [13]. Furthermore, a total number of 33 gene deletions affecting 7 genes were founds, whereof 27 (81.8%) could be validated. In an analysis of 16 normal tissues, gene amplifications were only detected in one of the samples, and gene deletions were detected in none of the samples supporting the notion that Ioncopy is a highly specific method for calling CNAs.

Gene deletions are usually associated with a lower change of CNs and more difficult to detect than gene amplifications. In AS data, CN are estimated from coverages of the amplicons and therefore the sensitivity to discriminate between CNs is related to the precision of the CN estimates. The precision of copy number estimation can be characterized by the width of the inter-sample CN distribution that harbored SDs between 0.20 and 0.60 for the majority (> 90%) of the amplicons. Gene deletions correspond to copy number changes ΔCN = 1 or 2, while gene amplifications can harbor much higher ΔCNs, for example 5 or even 10. Furthermore, gene deletions are more prone to false positive detection because technical problems may cause malfunction of particular amplicons resulting in low or zero coverages. Ioncopy addresses these differences by using different detection algorithms for gene amplifications and for gene deletions. Detection of a gene amplification was based on detection by a single amplicon and multiple testing correction of the p-value for samples and amplicons, In contrast, detection of a gene deletion was based on detection by at least four amplicons using raw p-values without multiple testing correction.

Analyzing the agreement between amplicons in exons 19, 20 and 21 of *HER2*, we observed very strong pairwise correlations (all R > 0.93) and an excellent agreement of CNA calls: 17 tumors (9.2%) were classified as *HER2* amplified by all three amplicons, 162 tumors (88.0%) were classified as *HER2* unamplified by all three amplicons, while disagreements occurred only for 5 tumors (2.7%). For *HER2* status determination using the 2013 ASCO-CAP guidelines [14] as gold standard, NGS based CNA calling had a sensitivity of 90.0% and a close-to-perfect specificity of 98.8% and an overall agreement of 97.8%. As discussed before, the NGS based method had excellent inter-amplicon reproducibility and NGS results showed a high degree of co-amplification among the genes *HER2*, GRB7 and STARD3 that are located close together in 17q12. Thus, the limited sensitivity for detection of *HER2*+ tumors is most likely not due to a lacking sensitivity of the NGS-based method but a consequence of one or more of the following factors: (i) Some of the tumors might be wrongly classified as IHC 3+ and thus *HER2+* being negative for *HER2* amplification in reality. (ii) According to the ASCO-CAP guidelines, a *HER2* test result has to be reported as positive if either *HER2* copy number signal ≥ 6.0 or HER/*CEP17* ratio ≥ 2.0. In principle, it would be possible that cases below the detection limit of Ioncopy (CN > 3.51 for the amplicon in exon 19 of *HER2*) are classified as *HER2*+ by SISH. (iii) CNAs are diminished by contamination with normal tissue and therefore below the detection limit. (iv) The same effect might be caused by subclonality of the tumor cells, where some tumor cells are *HER2*-amplified, but the majority of tumor cells is not amplified for *HER2*. (v) Inter-block tumor inhomogeneity may contribute to the discordance as NGS and IHC/SISH were not conducted using consecutive sections because we used frozen tissues for sequencing but FFPE tissues for *HER2* status determination. Interestingly, there was a perfect agreement for the tumors with high (≥ 11.75) *HER2* gene expression: all of them were *HER2*+ and all of them got called for *HER2* amplification by Ioncopy. This observation supports the view that a large portion of the discrepancy is caused by tissue inhomogeneity. It should be noted that the comparison of *HER2* status assessment at different laboratories and even between different pathologists at the same laboratory has been reported to have an agreement in the range of 67-92% [15]. For example, agreement on *HER2* status between local and central laboratories in the NCCTG N9831 trail was 85.8% [16]. Taking into account inter-laboratory and inter-pathologist variance, it is unrealistic to expect a perfect agreement of NGS and Ioncopy with the visual method. Moreover, Ioncopy results represent an average value over the tumor tissue used for sequencing whereas visual scoring is performed at a comparatively smaller region leading to variations due to sampling effects.

The new method for CNA detection has certain limitations. Firstly, as Ioncopy does not use paired normal tissues for CNA detection, it is not possible to distinguish between germline CNVs and somatic CNAs. However, although germline CNVs are much less frequent than acquired CNAs in most solid cancers, both of them can contribute to tumorigenesis and tumor growth. Thus, from the standpoint of clinics and of treatment options gene dose changes are important, but it may be unimportant if the change is hereditary or acquired. In this context, it can be considered as an advantage, that both kinds of changes are covered by a single analysis.

**Figure 5:**
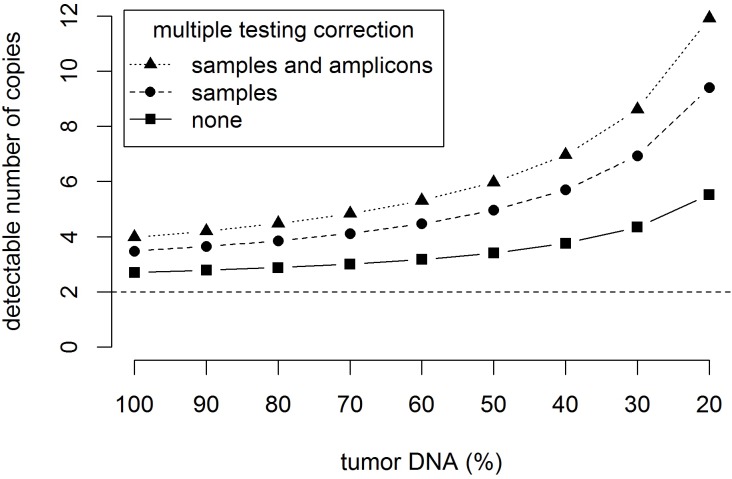
The effect of contamination with normal (germline) DNA on the detection limit for gene amplifications The graphics shows the number of gene copies that can be significantly (*p* < 0.05) separated from two gene copies. Detection limits are shown for an increasing contamination with normal DNA corresponding to a decreasing sample purity of 100%, …, 20% of tumor DNA. The results shown corresponds to a standard deviation sd = 0.43 of the copy number distribution and 184 samples and 152 amplicons under investigation.

Secondly, contamination of tumor DNA with normal DNA diminishes the degree of gene amplification or gene deletion detectable in the extracted DNA that is investigated by AS. Normal tissue contamination has two important implications: 1. The sensitivity to detect CNAs is diminished 2. Detected changes of CNs are less pronounced. Figure 5 shows the effect of contamination with normal (germline) DNA on the detection limits for CN gains: Using multiple testing correction for samples and amplicons, the detection limit increases from 3.99 via 4.48 and 5.97 to 8.62 when the tumor DNA content decreases from 100% via 80% and 50% to 30%. In cohorts with low tumor content, calling amplifications based on calls of more than one amplicon can help to lower the detection limit and to increase sensitivity. The second issue can be corrected *post hoc* using a linear transformation CN[tumor] = 1/TA × (CN[mixture] - 2) + 2 (TA = tumor area in %). In the cohort under investigation, these effects are moderate because the average tumor content was 85.7% in the investigated samples. However, it can be more pronounced in samples with less tumor content. *In situ* methods like FISH are more accurate in this context, but lack the opportunity of scalability and high-throughput investigation of many genes.

Thirdly, the algorithm is built on the assumption that the majority of tumors in the cohorts is unaltered at each genomic position under consideration. Under this assumption, the null distribution can be validly estimated using median and median absolute deviation. The assumption is fulfilled for most if not all of the genomic regions in many population-based cancer cohorts. In breast cancer, the most frequent CNAs affect only 5% to 20% of tumors in a representative population [6]. However, this assumption might be violated for some chromosomal regions in some tumor types. In this case, a cohort of normal tissues (or blood) can be sequenced and used as control. Ioncopy can be applied in such a way that normalization constants and thresholds for CNA calling are estimated in the normal tissue cohort.

We presented and evaluated a new algorithm to detect CNAs in AS data. Ioncopy offers the opportunity to call gene amplifications and gene deletions together with ”conventional” mutations such as point mutations, small insertions and deletions from the same data set. When designing a panel for conventional mutation and CNA analysis, we recommend including at least three amplicons for each gene that is investigated for amplifications and six amplicons for each gene that is investigated for deletions. A minimum number of one amplicon is needed for the detection of gene amplifications, while a minimum number of four amplicons is needed for the detection of gene deletions. The additional amplicons allow for internal validation and help to circumvent technical errors caused by possible malfunction of particular amplicons.

Targeted NGS has reached the status of a routine diagnostics application that is used to interrogate targetable simple somatic mutations in a tumor. Here, we extended its use to the calling of gene amplifications and gene deletions in a reproducible and reliable way. Implementation of gene amplification calling to together with somatic mutation calling in a single and easy-to-use assay is a step forward towards an intensification of research on the clinical implications of CNAs and implementation of suitable actionable CNA markers in routine diagnostics.

## MATERIALS AND METHODS

### Tumor cohort

The study cohort consisted of 184 fresh-frozen breast cancer tissues from a biobank at the Pathology Department of Charité Hospital. The project was approved by the ethics board of the Charité Hospital (Reference number EA1/139/05 Amendment 2008). Samples were included after passing histopathological quality control confirming ≥ 40% tumor area. The average tumor area was 85.7% (range 40% - 100%). *HER2* status was determined according to the 2013 ASCO-CAP guideline recommendations [14] using FFPE tumor tissues. Accordingly, *HER2*+ tumors were either immunohistologically positive (IHC 3+), harbored ≥ 6 signals/cell in single-probe SISH or harbored a *HER2*/*CEP17* ratio ≥ 2 in dual-probe SISH. Additionally, a cohort of 16 fresh-frozen normal breast tissues was analyzed.

### Sequencing panel design and targeted sequencing

A breast cancer specific sequencing panel of 154 amplicons was designed to cover the most important mutation hotspot regions and the most important gene amplifications of breast cancer. The panel included 48 genes of which 37 (77.1%) were interrogated by at least two amplicons: *AFF2*, *AKT1*, *APC* (3x), *ARID1A* (2x), *BRAF*, *CASP8* (2x), *CBFB* (2x), *CCND1* (3x), *CDH1* (13x), *CDK4* (2x), *CDKN1B* (2x), *CEP164*, *CTCF* (3x), *EGFR* (4x), *ERBB2* (3x), *ESR1*, *FGFR1* (2x), *GATA3* (3x), *GIGYF2*, *GRB7* (3x), *HERC1*, *KRAS* (2x), *MAP2K4* (5x), *MAP3K1* (11x), *MDM2* (3x), *MED1* (3x), *MLL3* (5x), *MYC* (3x), *NR1H2*, *PAK1* (3x), *PIK3CA* (6x), *PIK3R1* (3x), *PTEN* (6x), *PTPRD* (3x), *RB1* (11x), *RBMX*, *RPS6KA1* (3x), *RUNX1* (3x), *SF3B1* (2x), *STARD3* (3x), *TBL1XR1*, *TBX3* (2x), *TLR4* (3x), *TOP2A* (3x), *TP53* (7x), *TSHZ2* (3), *USP36* and *ZNF703* (3x). The mean amplicon length was 119 bp (min = 91 bp, max = 135 bp). Semiconductor sequencing [17] was executed using the Ion Personal Genome Machine (PGM) system (Thermo Fisher Scientific).

Preparation of total DNA from frozen breast cancer tissues were performed as follows: Ten consecutive 10 μm tissue sections were prepared, the first section was stained with hematoxylin/eosin and the tumor containing area was marked by a pathologist. DNA was extracted from the remaining nine sections using QIAamp DNA Mini Kits (Qiagen GmbH, Hilden, Germany) following the manufacturer's instructions. Total DNA concentrations were measured with Qubit fluorometer HS DNA Assays (Thermo Fisher Scientific, Waltham, MA, USA) and with TaqMan RNase P Detection Reagents Kits (Thermo Fisher Scientific). The final library was prepared starting from 10 ng of gDNA and quantified using qPCR (Ion AmpliSeq Library Kit 2.0 and Ion Library Quantitation Kit, Thermo Fisher Scientific). The Samples were 8-fold multiplexed and amplified on Ion Spheres Particles using the Ion OneTouch™ 200 Template Kit v2 DL (Thermo Fisher Scientific). After library enrichment and quality control on a Qubit instrument (Ion Sphere Quality Control Kit, Thermo Fisher Scientific), the samples were sequenced using the Ion 318 chip v2 according to the standard protocol of the chip manufacturer. Base calling and alignment to the human genome (hg19) were executed with the Torrent Suite Software 4.0.3.

### Whole genome expression data

Expression data were available for a subcohort of 111 tumors. Gene expression analysis was done as described before [18] using the cDNA-mediated Annealing, Selection, Extension, and Ligation (DASL) assay and the HumanRef-8 v3 Gene Expression BeadChip (Illumina, Inc., San Diego, CA).

### Calling copy number alterations

A new algorithm, Ioncopy, was developed to call CNAs from sequencing data of a cohort of tumors. The algorithm assumes that each of the genes is amplified or deleted in a minority of samples, while the majority of the samples harbors an unchanged gene dose of two alleles. The significance of CNAs is assessed by comparison with a null distribution that is estimated from outlier robust cohort estimates. Ioncopy was implemented using the statistical language R and is freely available from the CRAN repository (cran.r-project.org/package=ioncopy).

Starting from the BAM files of sequenced tumor DNA, the coverage of each amplicon in each sample is calculated by averaging over the sequencing depth at each base pair in the amplicon. The average GC content of the amplicons was 49% and varied from a minimum of 25% to a maximum of 82%. The average sequencing coverage was 4330 ± 1979 (mean ± sd). 152 out of 154 amplicons passed the quality control filter of harboring mean coverage ≥ 100 and were included in the analysis. Copy numbers (CNs) for each amplicon in each sample are estimated using a two-fold normalization: Firstly, for each of the samples, the coverage of each amplicon is divided by the median coverage of all amplicons in the sample. Secondly, for each of the amplicons, the coverage of each sample is divided by the median coverage of the amplicon in all samples and multiplied by two (to take two alleles into account). Estimated CNs (excluding outliers) turned out to be normally distributed in good approximation. Two methods are available to estimate the null distribution, “amplicon-wise” and “pooled”. For the former method, the SD of CN distribution was estimated individually for each amplicon using the outlier-robust median absolute deviation (MAD). For the latter method, the SD was estimated in the same way, but using the pooled distribution originating from all amplicons. Using the estimate from the pooled distribution, a MAD of 0.43 was obtained. A normal distribution with the estimated SD served as null distribution for significance assessment of CNAs. For the majority of amplicons (*n* = 140, 92.1%) the MAD of the CN distribution ranged between 0.20 and 0.60. However a few amplicons (*n* = 12, 7.9%) had considerably larger MADs (maximum = 1.25). To avoid an overestimation of the significance for the amplicons with larger intrinsic variation, we used the amplicon-wise method for the detection of CNAs in all analyses.

After calculation of the p-values, there were two kinds of multiple hypotheses testing that needed to be taken into account: Simultaneous testing for samples and simultaneous testing for amplicons. Ioncopy can be run in a mode without multiple testing correction (CNAs with low significance), a mode with multiple testing correction for the samples (significant CNAs) or in a mode with multiple testing correction for both samples and amplicons (highly significant CNAs). Using the pooled approach, gains with low significance (*p* < 0.05 without multiple testing correction) corresponded to CN > 2.70, significant (*p* < 0.05 after Bonferroni correction for 184 samples) gains corresponded to CN > 3.48, while highly significant gains (*p* < 0.05 after Bonferroni correction for 184 samples and 152 amplicons) corresponded to CN > 3.99. Losses with low significance corresponded to CN < 1.30, significant losses corresponded to CN < 0.52, while highly significant losses corresponded to CN < 0.01.

CN analysis of the normal tissue cohort was performed in the same way as the tumor cohort. Normal tissue data were internally normalized, but CNAs were called using the same threshold as in the tumor tissue cohort.

### Statistical analysis and graphics generation

Statistical analysis and generation of figures were executed using the statistical language R including the R packages multtest and beeswarm. All analyses performed for this paper (after calculation of the copy number matrix) took less than 5 minutes on an Intel Core i7-3820 CPU @ 3.60 GHz.

Significance assessment was executed with the method of amplicon-wise estimation of null distributions as described above. For the global analysis of gene amplifications, an alteration was considered as detected, when highly significant for at least one of the interrogating amplicons and as validated, when highly significant for at least one additional amplicon. Global analysis of gene deletions was performed without using multiple testing corrections. A deletion was considered as detected, when significant for at least four of the interrogating amplicons and as validated, when significant for at least one additional amplicon.

CNs of genes were calculated as average over the CNs of all interrogating amplicons. For clustering of tumors and genes, CNAs were represent as binary values (1 = amplified, 0 = normal). Hierarchical clustering was executed using the Manhattan distance to calculate the similarity between samples and the average linkage method to calculate the distance between clusters. The clustering diagrams were produced using the function heatmap from the R package stats.

The significance of gene expression changes between amplified (or deleted) tumors and unaltered tumors was assessed using Welch's t-test.
